# Occurrence of antibiotics and antibiotic resistance genes at various stages of different aquaculture modes surrounding Tai Lake, China

**DOI:** 10.3389/fmicb.2025.1543387

**Published:** 2025-01-31

**Authors:** Congcong Wu, Haitao Ye, Mingzhu Xu, Xuan Zhao, Xuejie Zhao, Lina Li, Mingzhi Li, Yanfei Wei, Yuru Li, Baolan Hu

**Affiliations:** ^1^Department of Environmental Engineering, College of Environmental and Resources Sciences, Zhejiang University, Hangzhou, China; ^2^Zhejiang Environmental Technology Co., Ltd., Hangzhou, China; ^3^Zhejiang Qingke Environmental Protection Technology Co., Ltd., Quzhou, China; ^4^Zhejiang Province Key Laboratory for Water Pollution Control and Environmental Safety, Hangzhou, China

**Keywords:** aquaculture modes, antibiotics, antibiotic resistance genes, microbial diversity, water pollution

## Abstract

**Introduction:**

Aquaculture is an important source of antibiotics and ARGs in environmental waters. However, the occurrence of antibiotics and ARGs under different modes and stages of aquaculture has rarely been systematically studied.

**Methods:**

This paper uses qPCR, LC-MS, and High-Throughput sequencing across different culture modes and stages to investigate antibiotics, resistance genes, and microbial communities in the water bodies, and analyze contamination differences between these modes.

**Results:**

The quinolone and chloramphenicol were the main antibiotics, and the highest absolute abundance genes were quinolone resistance genes (*qnrB*) and quinolone resistance genes (*sul1*), with the mobile genetic element (MGE) *intI1*, both of which exhibited a gradual seasonal increase. Microbial diversity also varies seasonally, especially with a gradual increase in the abundance of some pathogenic bacteria (*Flavobacterium*). Antibiotics and resistance genes were found at higher levels in fish ponds compared to shrimp and crab ponds, while they were lower in shrimp and crab ponds that utilized the ecological mode ponds than in the traditional culture mode ponds.

**Conclusion:**

Our study presents a comprehensive characterization of antibiotics and ARGs in aquaculture waters from various perspectives. Ecological aquaculture modes contribute to reducing antibiotic and resistance gene pollution in water bodies. These findings will support the optimization of aquaculture mode and antibiotic usage to the green and sustainable development of aquaculture finally.

## 1 Introduction

Aquaculture is one of the fastest-growing animal production sectors, contributes nearly 94 million tons of aquatic animal species, and supplies 15% of animal proteins in 2022 (FAO, 2024). To meet the increasing food demand from population growth, High-density stock farming has gradually become the mainstream of aquaculture. However, an associated issue is the excessive of antibiotics in aquaculture to promote growth and treat bacterial diseases (Santos and Ramos, 2016). A 2012 survey conducted by the Food and Agriculture Organization of the United Nations (FAO) found that oxytetracycline, florfenicol, and trimethoprim/sulfadiazine were the antibiotics most frequently used to control diseases on farms. Whether antibiotics are metabolized or not, they will enter the surrounding aquaculture environment, causing biological toxicity and the spread of antimicrobial resistance genes, ultimately threatening human health (Santos and Ramos, 2018). Actually, aquaculture wastewater is a significant source of antibiotics in the environment (Hossain et al., 2022).

China is the world’s largest producer and exporter of aquatic products and also involves extensive usage of antibiotics in aquaculture (Liu et al., 2017; Li et al., 2021). In 2017, China consumed 57.9% of the world’s antibiotics and produced 51.2% of global aquaculture production (Schar et al., 2020). Freshwater aquaculture is the primary aquaculture practice in China, predominantly conducted in ponds, consistently ranking first in aquaculture area and production. Owing to the high demand for water sources, freshwater aquaculture farms, characterized by numerous ponds, are commonly distributed around lakes or along rivers (Ministry of Agriculture and Rural Affairs of the People’s Republic of China, 2023). For instance, Zhejiang province, situated in the middle and lower reaches of the Yangtze River region, has a large number of fish ponds surrounding Tai Lake, contributing 30% of the province’s freshwater fish production (Zhejiang Provincial Bureau of Statistics, 2023). Recently, some studies have uncovered the distribution pattern of antibiotics in aquaculture water around Tai Lake (Song et al., 2016, 2017) and ARGs mainly in Tai Lake (Chen et al., 2019; Stange et al., 2019). However, there is limited available data on the contamination characteristics of ARGs and antibiotics, as well as their relevance with water quality and microbial diversity in various aquaculture practices and culture stages.

There are several different types of aquaculture systems, broadly classified as extensive or intensive production (Allen and Steeby, 2011). Examples of extensive aquaculture include Traditional pond culture, which can be further categorized as either monoculture (single species) or poly-culture (multiple species) (Egamberdiev et al., 2024). Conversely, intensive aquaculture like the In-Pond Raceway System (IPRS) requires precise monitoring of temperature, dissolved oxygen, and diet in high-density environments to improve fish yield (Schar et al., 2020). The main differences between the traditional model and the raceway system are culture density, condition control, and retention times. Raceway systems require a large amount of water exchange and short retention times, which may reduce the contact between microorganisms and antibiotics. Moreover, in the city of Huzhou in the north of Zhejiang province, there is a popular sustainable agriculture model that integrates mulberry cultivation, sericulture, and fish farming (Mulberry-dyke and Fish-pond System). Briefly, Mulberry leaves on the embankments feed the silkworms, whose waste and shed skin provide food for the fish in the pond, and the fish waste fertilizes the pond mud. It constitutes a sustainable recycled system that eliminates waste, reduces flood hazards, improves agricultural productivity, and purifies water (Li et al., 2011).

In general, various aquaculture practices may result in different levels of contamination. For instance, intensive aquaculture has relatively poor water quality, which tends to animal disease, thus requiring the use of different types and doses of antibiotics (Xu et al., 2020). Aside from the aquaculture modes, the impact of antibiotic contamination varies at different aquaculture stages due to the addition of antibiotics based on the health status of the organisms (Zhang et al., 2023). Therefore, we hypothesized that diverse aquaculture practices and stages could lead to varying contamination profiles of ARGs in terms of type and abundance. To confirm that, it is necessary to investigate and characterize the occurrence and spatiotemporal variation of antibiotics and ARGs across various aquaculture modes and stages throughout the entirety of the aquaculture cycle.

In the present study, three predominant aquaculture modes around Tai Lake in Zhejiang province (Traditional pond, In-Pond Raceway System, and Mulberry-dyke and Fish-pond System) were selected to investigate the antibiotics, antibiotics resistance genes, and microbial community present in the aquatic water of these modes, as well as to explore their correlations with each other. In addition, we analyzed antibiotics and resistance genes as well as microbial community changes in water samples at three aquaculture stages (non-aquaculture, early, and middle) to assess the impact of different aquaculture stages on the level of contamination. The results of this study could provide insights into the use of antibiotics in different modes and stages of aquaculture, and further understanding of antibiotic and ARGs contamination, as well as guide antibiotic use, contributing to the development of environmentally friendly, healthy, and sustainable aquaculture.

## 2 Materials and methods

### 2.1 Sampling strategy and site characterization

**Figure 1:**
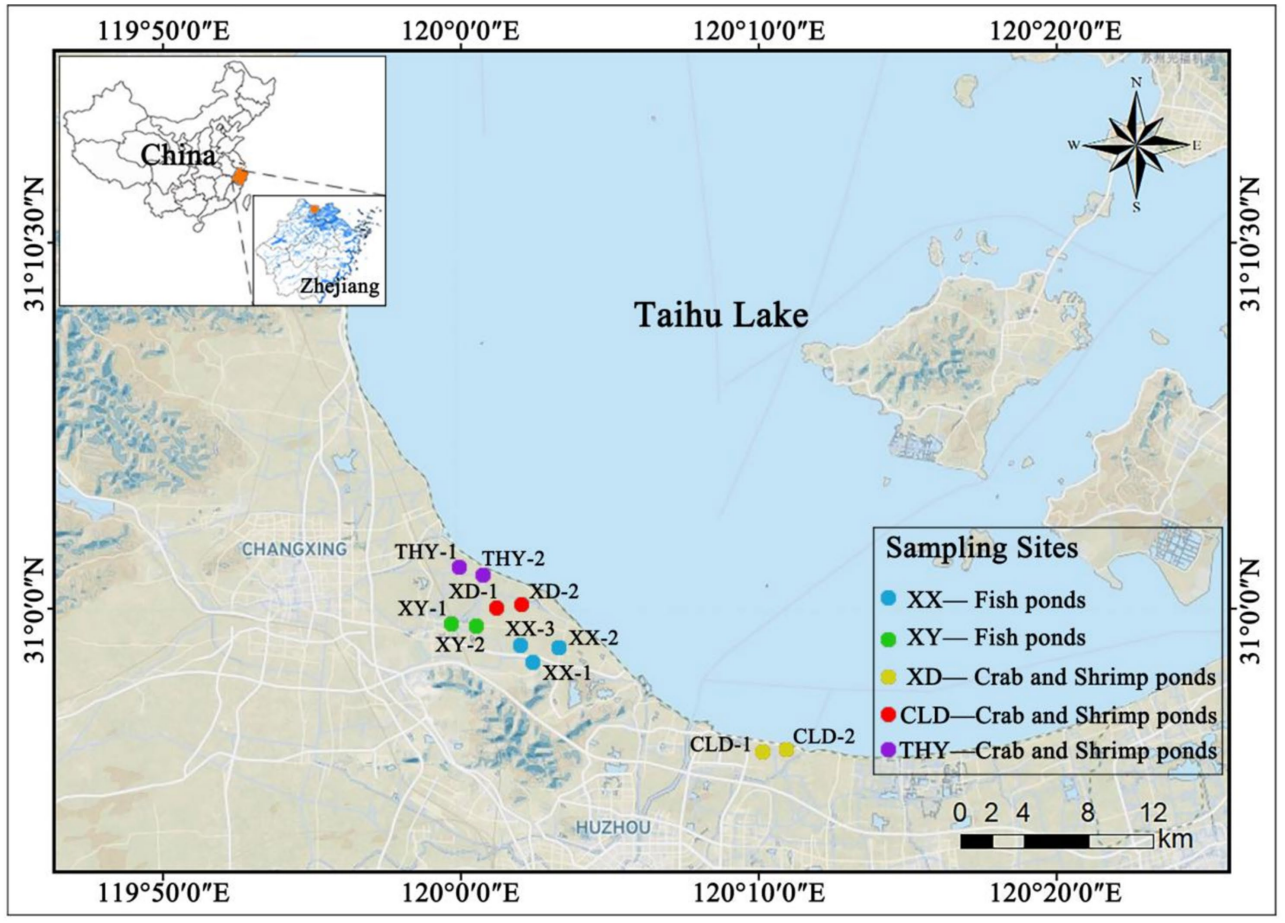
Sampling sites located in the southern region of Tai Lake, China.

This study investigated three aquaculture systems at sampling sites in the aquaculture areas around Tai Lake in Zhejiang Province, China (Figure 1). These systems consisted of Traditional ponds, a Mulberry fish pond, and an In-pond raceway system. The aquaculture practices in these systems included monoculture of Chinese mitten crab, yellow catfish, and shrimp, as well as poly-culture involving shrimp and other organisms (Supplementary Table S2).

Sampling occurred in January, March, and July 2024, representing the non-aquaculture stage, early, and mid-late stage, respectively Two liters of surface water samples (20–50 cm) were collected from five random points in each pond and then mixed in brown glass bottles. All samples were stored in a portable refrigerator and promptly transferred to the laboratory for subsequent processing.

### 2.2 Quantitative PCR of ARGs and microbial diversity assay

#### 2.2.1 DNA extraction

To extract ARGs from the water sample, the obtained water sample was first passed through a 0.22 μm sterilized membrane filter, with the resulting membrane subsequently stored at −80°C. Subsequently, total DNA was extracted from the membrane utilizing the Environmental DNA Extraction Kit (Simgen), following the manufacturer’s instructions. The quality of the extracted DNA was assessed by determining the absorbance ratio at 260 nm to 280 nm and by conducting agarose gel electrophoresis. Finally, the concentration of the DNA was quantified using the Synergy H1 Hybrid Multi-Mode Reader (Agilent, BioTek).

#### 2.2.2 Quantitative PCR of ARGs

The study employed qRT-PCR to measure the abundance of various antibiotic-resistance genes (ARGs). These included two sulfonamide resistance genes (*sul1*,*sul2*), six tetracycline resistance genes (*tetA*, *tetB*, *tetC*, *tetG*, *tetM,* and *tetW*), four quinolone resistance genes (*qnrA*, *qnrB*, *qnrS*, and *gyrA*), three chloramphenicol resistance genes (*floR*, *cmlA*, and *cat1*), three erythromycin resistance genes (*ermB*, *ermT*, and *ermF*), along with one mobile genetic element gene (*intI1*). *IntI1* is an important indicator for the spread of ARGs. These ARGs were chosen from those identified in aquaculture water in previous studies (Lu et al., 2019; Wang et al., 2022). All of the qPCR analysis was performed on QuantGene9600 (FQD-96C) fluorescent quantitative detection system (Bioer, China) using ChamQ SYBR qPCR Master Mix (Q321-02, Vazyme). The plasmid containing the target genes was employed as standard in the quantitative PCR designed by Wcgene Biotechnology Corporation (Shanghai, China).

The copy numbers of all the target genes were calculated based on the standard curve as the absolute gene abundance to assess the contamination level of ARGs in aquaculture water. The PCR reaction system and procedure for each gene were previously described (Xue et al., 2021; You et al., 2018). All qRT-PCR experiments were repeated three times. The details of genes and primers are shown in Supplementary Table S3.

#### 2.2.3 Microbial diversity assay

High-throughput sequencing of 16 s rRNA gene is used to investigate bacterial diversity. Sequencing services for environmental samples were conducted via Magigene Biotechnology Co., Ltd. (Guangzhou, China). The primer 341F (5’-CCTAYGGGRBGCA SCAG-3′) and 806R (5’-GGACTACNNGGGTATCTAAT-5′) were used for PCR amplification of the V3-V4 region of the bacterial 16S rRNA gene, and the 16S rRNA of samples was measured with the NovaSeq PE 250 platform. The MAGICHAND cloud platform analyzed all gene sequence data.

### 2.3 Water quality and antibiotics detection

Water quality was measured by using UV–VIS Spectroscopy UV-1900i (Shimadzu Corporation). The total nitrogen (TN), ammonia nitrogen (NH_4_^+^), nitrate (NO_3_^−^), nitrite (NO_2_^−^), phosphorus (TP), and chemical oxygen demand (COD) were detected according to the spectroscopic methods provided by the Standard Methods for Examination of Water and Wastewater (Eaton, 2005).

Based on the current status of antibiotic usage in aquaculture (Chen et al., 2017; Tong et al., 2014; Zhang et al., 2023; Zhang et al., 2021), 32 target antibiotics widely used to treat human and animal diseases were selected for detection in water samples. These antibiotics comprised 15 sulfonamides (SAs): Sulfadiazine (SDZ), Sulfisomidine (SDM), Sulfamethazine (SMZ), Sulfamerazine (SMR), Sulfamethoxazole (SMX), Sulfamono methoxine (SMM), and 11 additional sulfonamides; six fluoroquinolones (FQs): Ofloxacin (OFL), Norfloxacin (NOR), Ciprofloxacin (CIP), Enrofloxacin (ENR), flumequine (FL), and Oxilinic acid (OA); four macrolides (MLs): Tylosin (TYL), Clarithromycin (CTM), Azithromycin (AZI), and Roxithromycin (ROX); three Tetracyclines (TCs): Tetracycline (TC), Oxytetracycline (OTC), and Chlortetracycline (CTC); three Chloramphenicol (CAPs): Florfenicol (FFC), Chloramphenicol (CAP), and Thiamphenicol (TP); and one anthelmintic thiabendazole. The details of antibiotics are listed in Supplementary Table S4 of Appendix A.

Antibiotic extraction was conducted as previously described (Zhang et al., 2023). To describe briefly, water samples were pretreated with 5.0 mL of methanol and 0.50 g of EDTA-Na_2_ and then stood for 2 h. The pH was adjusted to approximately 4.0 using a 50% (V/V) aqueous phosphoric acid solution and the solid-phase extraction (SPE) column was pretreated with 5.0 mL of methanol followed by 5.0 mL of ddH_2_O. Pretreated water samples were then passed through the AutoTrace™ 280 SPE (Thermo Fisher, United States) at a flow rate of 4.0 mL/min. The final elution was performed sequentially with 4.0 mL of methanol and 4.0 mL of an ammonia-methanol solution (5: 95, v/v). The eluate was subsequently concentrated using nitrogen blowing (TurboVap®II, Caliper) and adjusted to a final volume of 1.0 mL with methanol. Finally, the concentrated solution was filtered through a 0.22 μm organic microporous membrane into a sample bottle. The samples were assayed for antibiotic content by liquid chromatography/mass spectrometry (LC–MS), and the specific conditions and parameters are detailed in Appendix Text S1.

### 2.4 Statistic analysis

The heat map was presented using TB tools (V2.070) (Chen et al., 2023). Homogeneity and Normality were checked using Levene’s test and Shapiro–Wilk test. The Tukey’s test or Kruskal-Wallis test was used to analyze the differences in antibiotic concentration and antibiotic resistance genes across various aquaculture practices and stages. The experimental data were analyzed and calculated using SPSS 20.0. Correlation heatmap analysis and visualization are conducted using Origin Pro 2024b. Mantel’s tests determined the correlations between microbial community composition and other antibiotics as well as water quality parameter. The R (version 4.4.0) package linkET was utilized for data analysis and visualized by the Online website tools ChiPlot^1^ (Ji et al., 2022).

## 3 Results

### 3.1 Absolute abundance and spatiotemporal distribution of ARGs

**Figure 2:**
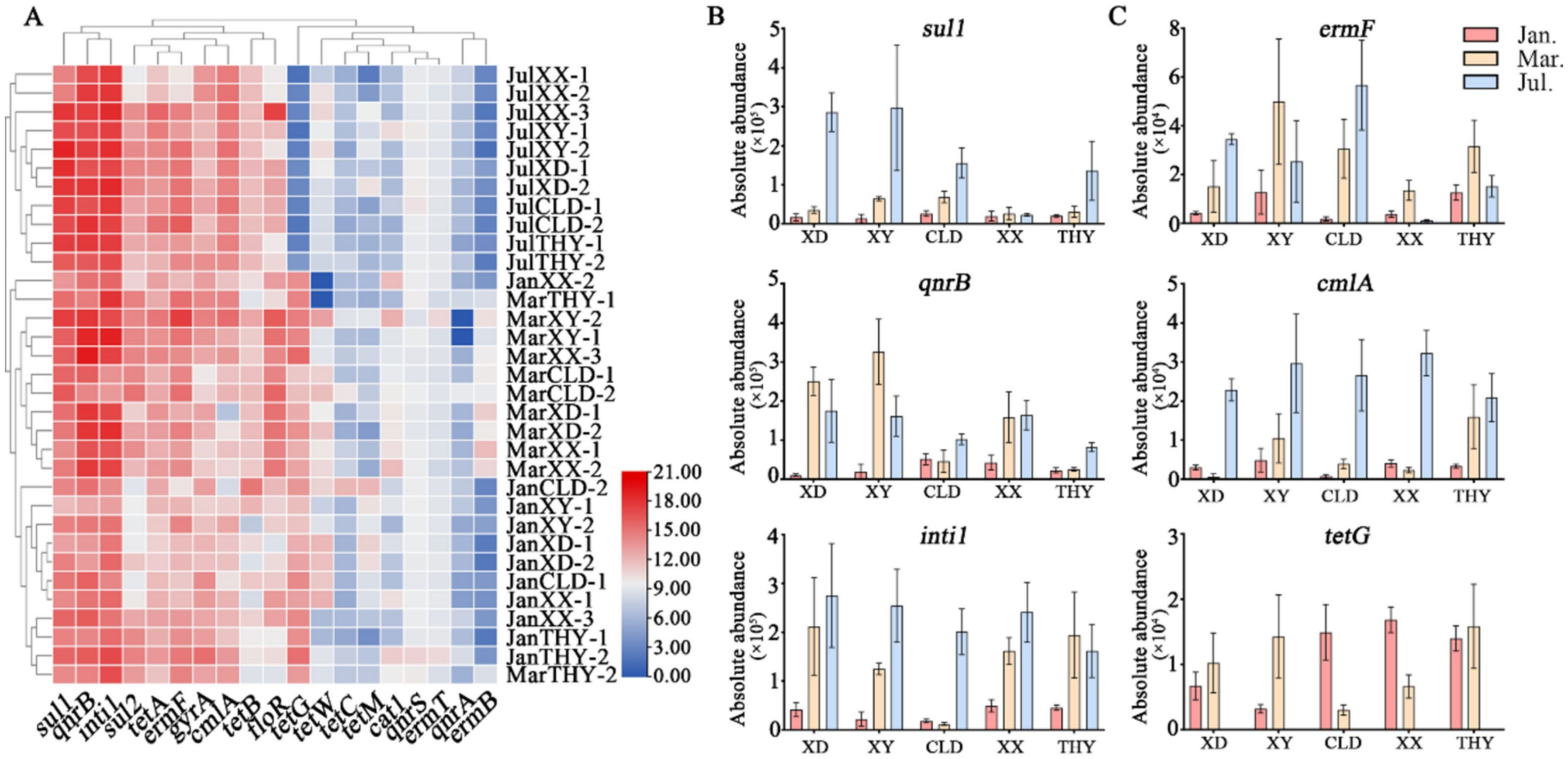
Spatiotemporal distribution and variations in absolute abundances of antibiotic resistance genes (ARGs) across five sites at three stages of aquaculture. **(A)**, Heatmap of expression levels for various resistance genes at five different locations (XD, XY, CLD, XX, and THY) across different stages. **(B)**, Spatial and seasonal variation in absolute abundance of *sul1*, *qnrB,* and *intI1* genes. **(C)**, Spatial and seasonal variation in absolute abundance of *ermF*, *cmlA*, and *tetG* genes.

The abundance of antibiotic resistance genes (ARGs) in aquaculture water at different modes and stages is illustrated in Figure 2. Notably, the overall absolute abundance of ARGs indicates that *tetA*, *tetG*, *sul1*, *qnrB*, *ermF*, and *intI1* are highly prevalent, while *ermB*, *tetC*, *tetM*, *qnrA*, *qnrS*, *cat1*, and *ermT* are consistently exhibited lower abundance across all sites and time (Figure 2A).

During the non-aquaculture stages in January, water from various ponds typically showed lower abundances of antibiotic resistance genes (ARGs). However, some target genes were still present in significant quantities. For instance, *sul1* (ranging from 4 × 10^3^ ~ 5.6 × 10^4^ copies/mL), *qnrB* (6.5 × 10^3^ ~ 7.8 × 10^4^), and *inti1* (9 × 10^3^ ~ 5.6 × 10^4^) were observed among all kinds of sampling sites (Figure 2B). In March, during the start of the breeding season, the data indicate a higher abundance of ARGs across various aquaculture sites compared to January. The two highest abundance genes, *qnrB* (7.2 × 10^4^ ~ 6.0 × 10^5^), and *intI1* (9.1 × 10^4^ ~ 3.1 × 10^5^), both reach a magnitude of 10^5^ at most of the sampling sites. The *sul1* gene still keeps a high abundance of 1.5 × 10^4^ ~ 1.9 × 10^5^ copies/mL, while the *sul2* (8.3 × 10^3^ ~ 3.6 × 10^4^), *gyrA* (1.6 × 10^3^ ~ 1.9 × 10^4^), *floR* (1.9 × 10^4^ ~ 4.0 × 10^4^), *tetA* (3.0 × 10^3^ ~ 2.0 × 10^4^), *tetB* (2.2 × 10^2^ ~ 7.4 × 10^3^), and *ermF* (5.9 × 10^3^ ~ 4.1 × 10^4^) genes increased significantly by an order of magnitude compared to earlier aquaculture stages. In July, the average absolute abundances of most target genes peak at all sample sites. Among them, the *sul1* (7.6 × 10^4^ ~ 4.4 × 10^5^) and *intI1* (1.2 × 10^5^ ~ 3.7 × 10^5^) genes remain highly abundant, maintaining their dominance as some of the most prevalent resistance genes. Seasonal trends were observed in most genes, particularly in *ermF* and *cmlA*. Specifically, *ermF* increased from (1.0 × 10^3^ ~ 5.0 × 10^3^) in January to (2.8 × 10^4^ ~ 3.5 × 10^4^) in July, and *cmlA* rose from (1.5 × 10^3^ ~ 4.0 × 10^3^) in January to (2.0 × 10^4^ ~ 4.5 × 10^4^) in July (Figure 2C). Interestingly, the tetracycline resistance gene, the *tetG* gene decreased from high abundance in January/march (10^3^ ~ 10^4^) to July (below 10^2^), while the abundances of *tetA* and *tetB* genes gradually increased (Supplementary Figure S1).

Although seasonal variations in ARGs trends were evident, significant differences in the average absolute abundance and types of some genes were also observed among different sites. These differences were influenced by diverse aquaculture practices and species. Fish ponds, including both raceway systems (XX) and traditional ponds (XY), generally exhibit higher levels of ARGs compared to shrimp/crab ponds in July, such as *tetA*, *qnrB*, *intI1*, *cmlA*, and *florR* (Figure 2; Supplementary Figure S1). The analysis reveals distinct patterns of gene dominance between the XX and XY modes of the same aquaculture species. Specifically, the total average absolute abundances of the quinolone resistance genes (*qnrB* and *gyrA*) and the chloramphenicol resistance gene (*cmlA*), were significantly higher in the IRAS (XX) mode compared to XY (Figures 2B,C). In contrast, the sulfonamide resistance genes (*sul1* and *sul2*), macrolide (*ermF*), and tetracycline (*tetA*) resistance genes were more abundant in the XY mode than in XX. These results may suggest a variation usage of antibiotics between different practices. The trend for crab and shrimp ponds (XD, CLD, THY) also varied, with XD and CLD representing traditional ponds and THY representing an ecological system. The types, abundance, and seasonal trends of resistance genes such as *sul1*,*ermF*, *cmlA*, *intI1*, *tetA*, and *floR*, were similar in XD and CLD. In contrast, most resistance genes were significantly less abundant in THY compared to the other two sites (Figure 2; Supplementary Figure S1).

### 3.2 Antibiotics

**Figure 3:**
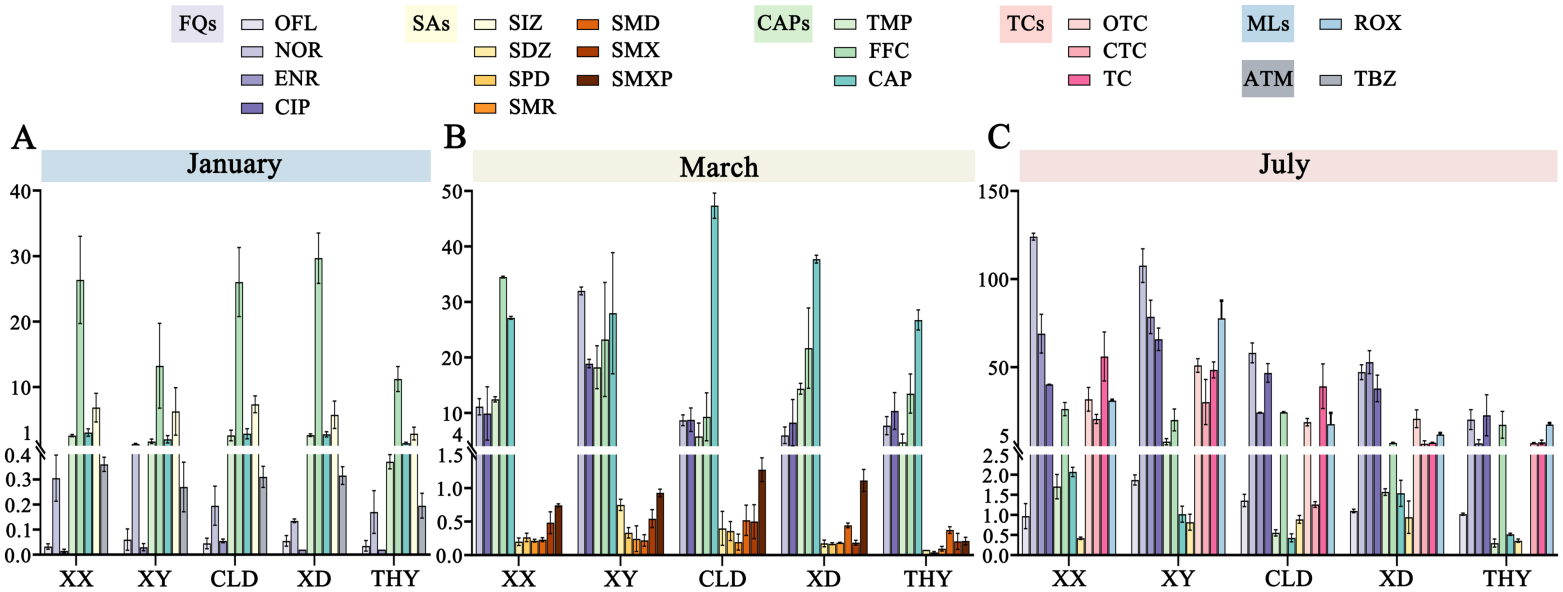
Spatiotemporal distribution and variations in the concentrations of detected antibiotics across five sites at three stages of aquaculture **(A-C)**. FQs, Fluoroquinolones; SAs, Sulfonamides; CAPs, Chloramphenicol; TCs, Tetracyclines; MLs, Macrolides; ATM, Anthelmintic.

In the southern region of Tai Lake, 31 antibiotics and one anthelmintic drug were analyzed in aquaculture ponds at five sites across three different stages. The overall variety of antibiotics in this region is similar. The predominant classes identified were quinolones, chloramphenicol, and sulfonamides, whereas tetracyclines and macrolides were detected only in July. The antibiotics with the highest concentrations included the quinolones norfloxacin, ciprofloxacin, ofloxacin, enrofloxacin, as well as the chloramphenicol analog florfenicol and thiamphenicol (Figure 3).

With the start of aquaculture, the concentration of all antibiotics ranged from not detected (n.d.) to 32.39 ng/L, with only eight antibiotics detected from 32 target antibiotics in all ponds. The highest concentrations of detected antibiotics were FFC (9.87 ~ 32.39 ng/L), SIZ (2.19 ~ 8.83 ng/L), CAP (1.13 ~ 3.44 ng/L), and TMP (0.39 ~ 3.16 ng/L). In contrast, the concentrations of OFL, NOR, ENR, and thiabendazole were lower, ranging from 0.01 to 0.5 ng/L (Figure 3A). In the breeding stage of March, both the concentration and types of detected antibiotics were significantly increased, with concentration ranging from n.d. to 48.97 ng/L. In this stage, chloramphenicol antibiotics were predominant, with concentration rising from n.d. in January to 20.25 ~ 48.97 ng/L in March. Meanwhile, FFC and TMP sustained high concentrations, ranging from 6.29 ~ 34.61 ng/L and 4.07 ~ 20.97 ng/L, respectively, and followed by quinolones CIP (5.33 ~ 19.43 ng/L) and NOR (4.93 ~ 32.48 ng/L) (Figure 3B). Particularly, several antibiotics of the sulfonamides such as SDZ, SPD, SMR, SMD, SMX, and SMXP were detected. However, the concentration was lower maintained at 0.1-1 ng/L. At the late aquaculture stage in July, Most detected antibiotics kept a higher concentration in the pond’s water bodies. The predominant antibiotics still were chloramphenicol and quinolone, such as FFC, CIP, ENR, and NOR, with concentrations ranging from 5.19 ~ 129.2 ng/L. Additionally, we were the first to observe erythromycin, a macrolide antibiotic, as well as oxytetracycline, chlortetracycline, and tetracycline belongs the tetracycline group. The concentration of these antibiotics ranges from n.d. to 73.93 ng/L. In contrast, only sulfadiazine among the sulfonamides was detected at very low concentrations (0.33 ~ 1.23 ng/L; Figure 3C). In summary, we observed a gradual increase in antibiotic concentrations and types from January to July.

The spatial distribution of antibiotics is also characterized. Based on the analysis of antibiotic concentrations in ponds of different species, revealed that fish ponds generally had higher antibiotic levels than shrimp and crab ponds. For example, in March, the concentrations of FFC, TMP, and CIP in fish ponds were 15.99 ~ 38.23 ng/L, 12.05 ~ 20.97 ng/L, and 6.51 ~ 19.43 ng/L, respectively, which were significantly higher than those of the corresponding antibiotics in shrimp and crab ponds (FFC: 6.26 ~ 26.79 ng/L; TMP: 4.07 ~ 15.06 ng/L; CIP: 5.33 ~ 12.71 ng/L). Aquaculture modes and the type of culture species influence the spatial distribution of antibiotics. Most antibiotic concentrations in shrimp and crab ponds under the ecological culture model were lower than those in traditional ponds. The same trend is also observed in July. Likewise, antibiotic concentrations in the fish ponds of the strictly controlled raceway system were also lower than those in conventional culture modes (Figure 3).

### 3.3 Microbial diversity

**Figure 4:**
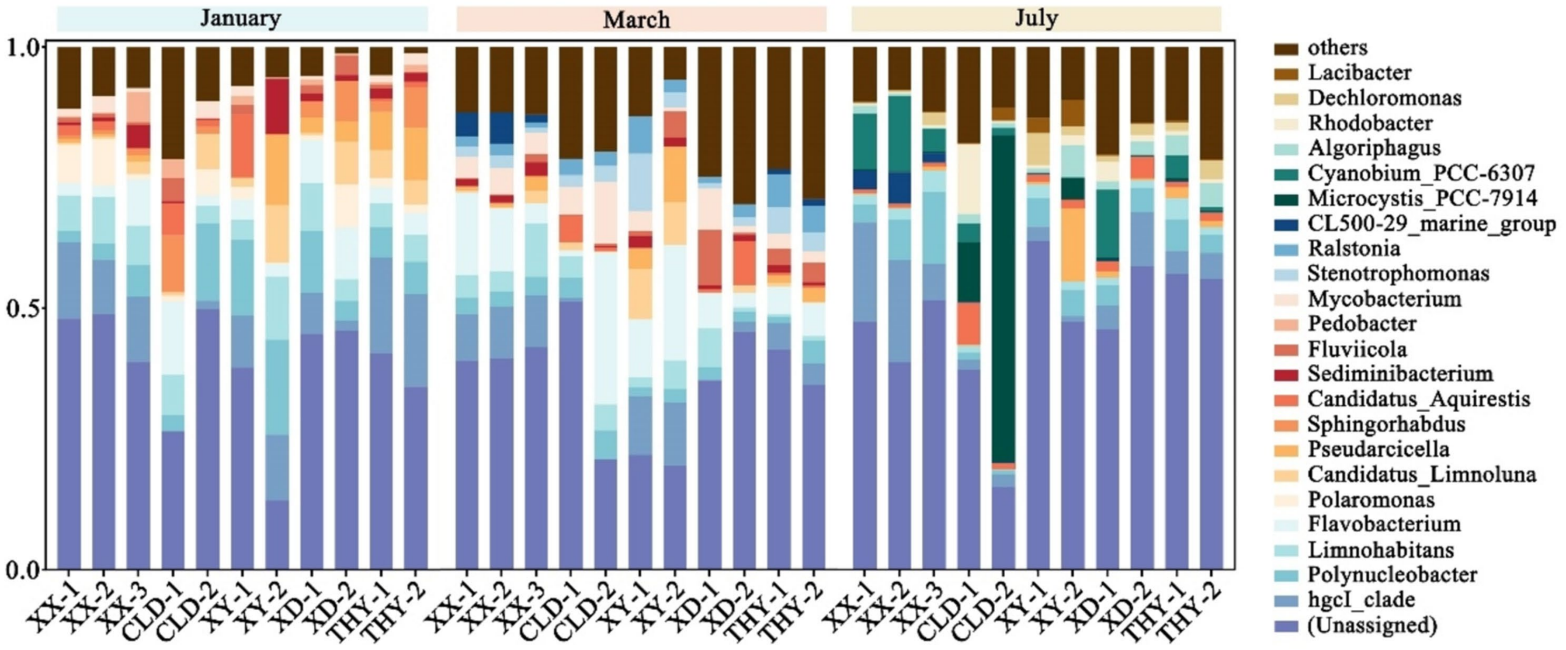
The bacterial genera with the highest relative abundances in each kind of aquaculture ponds in January, March, and October.

The microbial diversity in aquaculture ponds with different modes and seasons was analyzed. The predominant abundances of bacterial phyla found at all sampling sites were Proteobacteria, Actinobacteriota, Bacteroidota, Cyanobacteria, and Verrucomicrobiota (Supplementary Figure S2). Further research on microbial diversity revealed that the species composition and abundance of the genus in all ponds varied seasonally but were not influenced by spatial variation. In January, the highest abundance of the genus were hgcI_clade, Polynucleobacter, Limnohabitans, Flavobacterium, and Polaromonas. At the start of aquaculture in March, Flavobacterium, hgcI_clade, Limnohabitans, Mycobacterium, and Stenotrophomonas were the top five highest genus in all ponds. In July, the main five genera of microbial were hgcI_clade, Microcystis_PCC-7914, Cyanobium_PCC-6307, Polynucleobacter, and Algoriphagus. Notably, the abundance of Flavobacterium considered an important fish pathogenic bacteria gradually increases with aquaculture activity and there is a risk of fish diseases. Additionally, the increased abundance of the blue and green algae genera Microcystis_PCC-7914 and Cyanobium_PCC-6307 in July may be linked to the heightened risk of algal outbreaks during high summer temperatures (Figure 4).

### 3.4 Correlations between antibiotics, ARGs, and water quality

**Figure 5:**
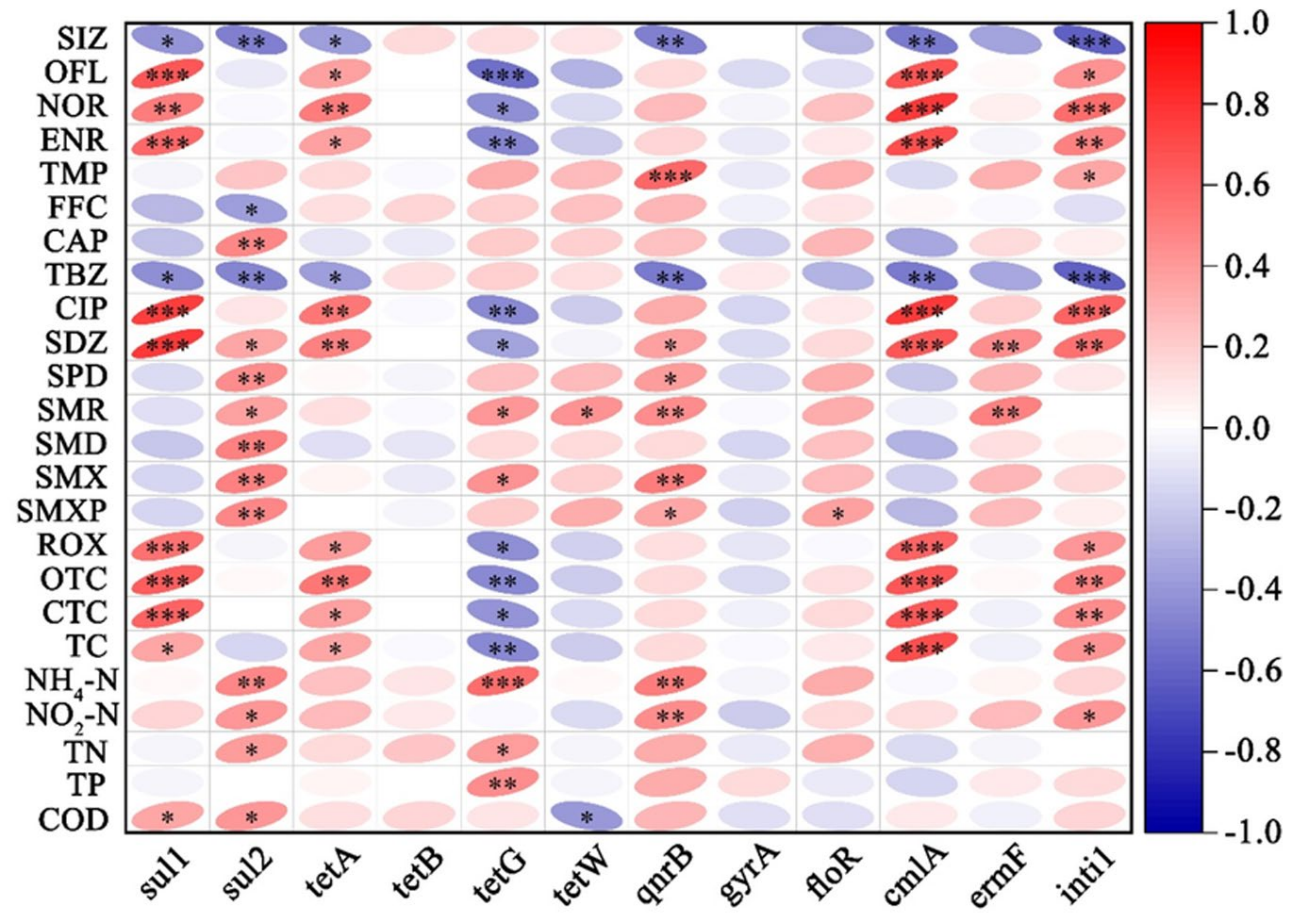
Correlation matrix between antibiotics, ARGs, and environmental factors in aquaculture water bodies. Red color indicates positive correlations and blue represents negative correlations. The intensity of the color reflects the strength of the correlation. Significance levels are denoted by asterisks, denoting different levels of statistical significance (* *p* < 0.05, ** *p* < 0.01, and *** *p* < 0.001).

We selected 11 main resistance genes with one MGE and examined their correlations with detected antibiotics and five water quality parameters. Correlations between the antibiotics, ARGs (including MGE), and water quality were illustrated in (Figure 5). For antibiotics, tetracycline (OTC, CTC, and TC), ROX, SDZ, and fluoroquinolones (OFL, NOR, ENR, and CIP) showed very significant positive correlations (*p* < 0.001 and *p* < 0.01) to the *sul1*, *cmlA*, and *intI1*, and significant positive correlations (*p* < 0.01 and *p* < 0.05) to the *tetA*, while negative correlations to *tetG* (*p* < 0.01 and *p* < 0.05). However, SIZ and TBZ showed significant negative correlations to the *sul1* (*p* < 0.05), *sul2* (*p* < 0.01), *tetA* (*p* < 0.05), *qnrB* (*p* < 0.01), *cmlA* (*p* < 0.01), and *intI1* (*p* < 0.001). In addition, the *Sul2* gene exhibited a strong correlation with most sulfonamide antibiotics (SDZ, SPD, SMR, SMD, SMX, and SMXP). For water quality, the correlation between ammonia (NH_4_) and various antibiotic resistance genes (ARGs), such as *sul2*, *tetG*, and *qnrB*, is significant positive. Similar to ammonia, nitrite shows positive correlations with *sul2* and *qnrB.* COD (chemical oxygen demand) showed significant positive with *sul1* and *sul2*. We also employed the Mantel test to analyze the relationship between microbial communities, antibiotics, and water quality, as illustrated in Supplementary Figures S3, S4. The results indicated that the microbial phylum composition is significantly correlated to most antibiotics, especially OFL. SDZ, CIP, CAP, and OFL significantly influenced microbial diversity at the genus level. Some antibiotics frequently utilized in aquacultures, such as FFC, ROX, SMX, SIZ, SPD, and CTC did not significantly influence microbial communities (Supplementary Figure S3). The correlations between bacterial community and water quality showed that nitrite (NO_2_-N) was significantly correlated with microbial diversity at the genus level. Water temperature significantly influenced the community changes of microorganisms by creating optimal growth conditions (Supplementary Figure S4).

## 4 Discussion

### 4.1 Absolute abundance and spatiotemporal distribution of ARGs

This study detected 5 types of ARGs, consisting of 18 subtypes and one MGE (Class I integron, *intI1*), which exhibited diverse absolute abundances among different water bodies. Overall, quinolones (*qnrB* and *gyrA*), sulfonamides (*sul1* and *sul2*), chloramphenicol (*cmlA* and *floR*), macrolide (*ermF*) resistance genes (ARGs), and *inti1* (MGE) were the dominant genes with high absolute abundances in different aquaculture month. However, quinolones (*qnrA* and *qnrS*), tetracycline (*tetC*, *tetW*, and *tetM*), and macrolide (*ermB* and *ermT*) resistance genes were observed at the lowest absolute abundances. This suggests that resistance to quinolones, sulfonamides, chloramphenicol, and macrolide was the predominant antibiotic resistance observed at aquaculture sites in this region. *IntI1* gene is known to capture and integrate ARGs into bacterial genomes, promoting the spread of resistance within bacterial communities. The abundance of *IntI1* in water bodies of different culture showed an increasing trend at different stages, indicating that aquaculture activities exacerbate the spread of resistance genes.

In this study, it is necessary to focus on the differences in abundance and variability between subtypes of antibiotic resistance genes (ARGs) within the same types. For instance, the absolute abundances of the tetracycline antibiotic efflux genes (*tetA*, *tetB*, and *tetG*) exhibited contrasting trends across samples from different seasons. The abundance of *tetA* and *tetB* gradually increased with aquaculture, whereas *tetG* decreased. This phenomenon may be related to the change in tetracycline antibiotics, water temperature, pH, or nutrient availability, which could favor bacteria carrying *tetA* and *tetB* over those with *tetG*, resulting in a shift in gene prevalence. We also found that the absolute abundances were observed as *qnrB* > *gyrA* > *qnrA* > *qnrS* in quinolone resistance, *ermF* > *ermT* > *ermB* in macrolides resistance, and *cmlA* > *floR* > *cat1* in chloramphenicol resistance. Studies have reported that the *qnr* gene is usually found in multiresistance plasmids associated with other resistance determinants (Jacoby et al., 2014). These mobile genetic elements facilitate horizontal gene transfer between bacteria, potentially contributing to a greater spread of *qnrB* compared to *gyrA*. Among the *qnr* gene family, the lower abundance of *qnrA* and *qnrS* may be due to the effect of specific antibiotic species. Erm genes family (ermB, ermF, and ermT) preventing macrolide binding to the ribosome and thus inhibiting their antibacterial action (Pechère, 2001). Differences in the abundance of these three genes may be due to the type and frequency of antibiotics, microbial community, and environmental pressures. The resistance mechanism of *cmlA* and *floR* gene were chloramphenicol efflux pumps and *cat1* encodes a chloramphenicol acetyltransferase that inactivates the antibiotic. *cmlA* is disseminated on transferable plasmids, contributing to its higher abundance (Bischoff et al., 2005). Furthermore, florfenicol is widely used in aquaculture due to its effectiveness in preventing and treating Vibrio infections. *floR* was the main epidemic resistance gene of florfenicol (Li et al., 2020), which contributes its high abundance. In summary, the abundance of different subtypes is determined by antibiotic selection pressure and propagation of these genetic elements. The high prevalence of resistance genes is generally influenced by the frequent usage of their corresponding antibiotics and the efficient modes of transmission of these genes (Yuan et al., 2019). Thus, investigations of resistance genes for multiple subtypes should consider antibiotic usage and the mode of gene transmission.

Different aquaculture modes are highly likely to exhibit distinct resistance genes due to variations in antibiotic usage, population density, and aquatic organism’s species (Xu et al., 2020; Zhang et al., 2021). However, limited research has compared the difference in resistance genes across various models within uniform regional conditions. In the present study, the absolute abundance of most ARGs was higher in fish ponds than in shrimp and crab ponds. These results are similar to previous studies in other regions (Wang et al., 2022; Xiong et al., 2015). In fish ponds, ARGs differ significantly between raceway aquaculture and traditional pond culture. The levels of *qnrB*, *gyrA,* and *cmlA* were elevated in runway culture (XX), while *sul1*, *sul2*, *ermF*, and *tetA* were more prevalent in traditional pond culture (XY). Raceway aquaculture (XX) exhibits greater water mobility and more frequent water exchange (Lutz, 2024), while traditional pond culture (XY) involves more sedentary waters. High-frequency water exchange can lead to shorter contact times between resistant bacteria and antibiotics, thus selectively retaining some ARGs to specific antibiotics. In contrast, static water conditions allow antibiotics to persist for extended periods increasing the selective pressure on certain antibiotics. This may explain the difference in types and abundance of ARGs between the two aquaculture modes. Among the shrimp and crab ponds, the types and abundance of ARGs were similar between the two traditional ponds (XD and CLD), while the ecological culture ponds (THY) exhibited a significantly lower level of ARGs than the other two. Other research also indicates that the ecological mode of rice-crayfish co-culture has lower potential risks of ARGs (Ning et al., 2022), suggesting that a sustainable ecological model is an effective strategy for mitigating ARGs.

In our present study, we observed significant seasonal variations in the abundance of resistance genes, including *qnrB*, *cmlA*, *sul1*, *intI1*, and *ermF* in both fish and shrimp/crab ponds, suggesting that seasonal factors or aquaculture activities may influence the level of ARGs. These genes increased by 1 to 2 orders of magnitude in July compared to January. Conversely, some genes exhibited a notable decrease in abundance during the same period, which suggests a change in selective pressures affecting these genes. Three sampling months represent distinct aquaculture stages with different antibiotic requirements, leading to changes in selective stress, which may have contributed to the seasonal increase in the abundance of ARGs (Lin et al., 2023). Therefore, providing appropriate antibiotic use guidelines for different modes and stages of aquaculture is essential to control the prevalence of ARGs.

### 4.2 Occurrence and disruption of antibiotics

The dominant antibiotics found in the southern region of Tai Lake were quinolones, chloramphenicol, and sulfonamides, particularly FFC, TMP, NOR, EOR, CIP, and OFL. Notably, the FFC, TMP, and NOR were detected at a frequency of 100% in fish, shrimp, and crab ponds throughout all sampling periods, indicating that they were frequently used in this region. Tetracycline and macrolide antibiotics were detected only in July, likely due to the higher temperatures during that month, which may compromise fish immunity and lead to increased disease susceptibility as well as consequently increase the use of these antibiotic groups (Franke et al., 2024). These results may explain the observed increase in the absolute abundance of *tetA*, *tetB*, and *ermF* in previous analyses of ARGs. The characterization of antibiotic use in aquaculture may be linked to the regulations on antibiotics imposed by the Chinese government. For example, chloramphenicol and oxytetracycline were replaced by florfenicol, a second-class veterinary drug approved by the Ministry of Agriculture of China in 1999 (Liu et al., 2017; Mo et al., 2017). Another class of quinolone antibiotics is a synthetic antibiotic that is widely used in aquaculture due to their strong antibacterial activity against Gram-negative bacteria, the main bacterial pathogens in aquaculture (Wang et al., 2022). We also found that the concentration of these detected antibiotics in this study was lower than in previous research (Song et al., 2016; Zhang et al., 2023), which may be attributed to different habits, aquaculture practices, and species in different regions. The seasonal dynamics in the concentration of antibiotics exhibited a gradual increase, yet some sulfonamide antibiotics disappeared. This trend of decreased types but higher concentrations aligns with results from previous studies (Zhang et al., 2023). We speculated that the reduced types of antibiotics during the middle-late aquaculture stage in July may result from high temperature and intense light, which enhance microbial activity and promote the photodegradation and biodegradation of antibiotics (Guo et al., 2024). Interestingly, most sulfonamide antibiotics keep a lower concentration in ponds at three sampling periods, yet the absolute abundance of the corresponding resistance genes *sul1* and *sul2* remains high. This phenomenon could result from two complementary resistance strategies – degradation and antibiotic resistance genes (ARG) – used by the microbial community. It has been reported that *in situ* degradation is predominant at high antibiotic concentrations, while the spread of antibiotic resistance genes (ARGs) plays a more significant role at lower concentrations (Vila-Costa et al., 2017).

In this region, the types of antibiotics in fish ponds were the same as in shrimp/crab ponds, while the concentration of most antibiotics in fish ponds was higher than in shrimp/crab ponds. This result is similar to the research in the aquaculture ponds surrounding the northern Tai Lake (Zhao et al., 2024). Furthermore, antibiotic concentrations in ponds with ecological culture mode are lower than those in traditional ponds, suggesting that an appropriate ecological aquaculture model can mitigate the pollution of antibiotics in water.

In conclusion, the results indicate that governmental management of antibiotics, the concentration of antibiotics used, and aquaculture practices significantly influence antibiotic contamination levels and the prevalence of resistance genes. Therefore, it is essential to legislate appropriately for antibiotic usage and to encourage ecological aquaculture methods to mitigate antibiotic pollution.

### 4.3 Microbial community

The overall phylum of microorganisms is similar to previous studies, suggesting that these phylum predominate in most freshwater aquaculture (Qin et al., 2024; Wang et al., 2022). For example, the hgcl_clade is abundant and widely distributed in our sampling sites. The hgcl_clade is typically found in freshwater habitats and is associated with nitrogen-rich environments (Gao et al., 2017; Ghylin et al., 2014). In the present study, seasonal dynamics in the microbial community were significant, especially in March. In the early stages of growth, the immune systems of fish and shrimp are not yet fully developed and are susceptible to the attack of various pathogens (Bedekar and Rajendran, 2022; Soto Dávila et al., 2020). The abundance of Flavobacterium and Stenotrophomonas were significantly increased compared to January. Flavobacterium species are significant pathogens affecting both wild and cultured fish globally. They can lead to many fish diseases such as gill damage, *skin* lesions, and deep necrotic ulcerations (Kumru et al., 2020). We also observed the emergence of the genus Stenotrophomonas, known for its intrinsic antibiotic resistance (Brooke, 2021), suggesting that may be related to the rise in antibiotic use at this stage. Additionally, In July, algae densities rose in some ponds, leading to observable algal outbreaks. This increase was reflected in microbial diversity, evidenced by a significant rise in the abundance of two genera Microcystis_PCC-7914 and Cyanobium_PCC-6307 from the blue and green Algae. In summary, aquaculture activities contribute to an increase in the abundance of pathogenic bacteria, which elevates the risk of fish diseases. To mitigate this risk, the use of antibiotics has risen significantly and this escalation in antibiotic use may explain the rapid increase in antibiotic concentrations observed in this study in March.

### 4.4 Correlations between antibiotics, ARGs, and water quality

The results reveal an interesting correlation between antibiotics and their corresponding ARGs. For example, the *sul2* gene exhibited a positive correlation with most sulfonamide antibiotics, whereas sul1 displayed a negative correlation with the corresponding antibiotics. However, the presence of *sul1* was strongly positively correlated with antibiotics from fluoroquinolone and tetracycline. The variation in transmission modes may explain the differences between *sul1* and *sul2, sul1* is associated with other resistance genes in class 1 integrons, while *sul2* is typically found on small nonconjugative plasmids or large transmissible plasmids (Antunes et al., 2005). Similarly, the three tetracycline efflux pump resistance genes showed different correlations among *tetA*, *tetB*, and *tetG*. These differences may result from variations in their mechanism, efficiency, and distribution. The *cmlA* gene showed a strong positive correlation with fluoroquinolone and tetracycline antibiotics, similar to *sul1* and *intI1*, and it is likely that class 1 integrons frequently link *sul1* and *cmlA*. Excess ammonia and nitrite damage fish gills, increasing stress and susceptibility to bacterial infections and other pathogens (Boyd, 2014). This higher vulnerability may lead to increased antibiotic use, potentially resulting in a rise in ARGs. COD, an indicator of organic pollution in water, shows positive correlations with several ARGs like *sul1* and *sul2*. Higher organic may facilitate the survival and growth of bacteria carrying these resistance genes. Additionally, suitable water temperature promotes the growth of microorganisms carrying ARGs and may also be responsible for the increased abundance of resistance genes. The community dynamics of microorganisms are significantly influenced by antibiotics, while some frequently used antibiotics such as FFC and CTC in aquaculture may lose their effectiveness in inhibiting microorganisms due to prolonged use.

## 5 Conclusion

This study systematically analyzes the spatial–temporal variations of antibiotics, ARGs, and microbial communities in water at three stages in five ponds based on three different culture models. We found that the main antibiotics used in this region’s aquaculture were quinolones, chloramphenicol, and sulfonamides, followed by tetracyclines and macrolides. Meanwhile, the higher abundant resistance genes were *sul1*, *qnrB*, and *intI1* (MGE), respectively. Temporal variation of antibiotics and ARGs were influenced by the aquaculture modes and species, these contaminations were found with higher abundances in fish ponds compared with shrimp/crab ponds, while same-species ponds utilizing ecological aquaculture modes showed lower concentrations of these pollutants in water than those in other modes. Seasonal variations at different culture stages reveal a clear trend: concentrations and types of antibiotics as well as the abundance of ARGs gradually increase. We also found an abundance of some pathogenic bacteria such as Flavobacterium gradually increased with aquaculture activity. Moreover, changes in the concentration and type of antibiotics influence the subtypes of the same class of antibiotic resistance genes, and lower concentrations of antibiotics can instead facilitate the spread of some ARGs. In conclusion, this study provides new insights into the occurrence of antibiotics and ARGs in different aquaculture modes and stages and further suggests that appropriate ecological modes and antibiotic use strategies can contribute to the prevention and control of new contaminants in aquaculture.

## Data Availability

The raw sequencing data of microbial communities have been uploaded to the NCBI Sequence Read Archive (SRA) under BioProject accession number: PRJNA1214510.

## Footnotes

^1^ https://www.chiplot.online/
