# Supplementary material for: Occurrence of antibiotics and antibiotic resistance genes at various stages of different aquaculture modes surrounding Tai Lake, China
Source: Front Microbiol. 2025 Jan 31;16:1543387. doi: 10.3389/fmicb.2025.1543387 (PMC11825748; doi:10.3389/fmicb.2025.1543387)
Supplement: Supplementary file 1 [file Data_Sheet_1.docx]

**Supplementary information**

**Text S1:** Analysis method of antibiotics.

**Table S1:** The gradient elution procedure of LC-MS for antibiotics analysis.

**Table S2:** Information of aquaculture for five sampling ponds.

**Table S3:** qPCR primers and reaction conditions for antibiotic resistance gene detection.

**Table S4:** All antibiotic types tested in this study.

**Figure S1:** Spatiotemporal distribution and variations in absolute abundances of additional ARGs.

**Figure S2:** The bacterial phyla with the highest relative abundances in each kind of aquaculture ponds in three stages.

**Figure S3:** The correlation between bacterial community (genus and phylum ) and antibiotics.

**Figure S4:** The correlation between bacterial community (genus and phylum ) and water quality.

**Text S1** Analysis method of antibiotics

All analytical antibiotic standards and isotope internal standards were purchased from J&K Scientific (Beijing, China), and the purity was >99.0 %. antibiotic standards were prepared as a standard stock solution of 1000 mg/L in methanol, and then the standard stock was diluted to a mixed standard solution of 10.0 mg/L and stored at -20°C for use.

Target antibiotics analysis was performed on the Agilent 1290Infinity II ultra-high-performance liquid chromatography (UHPLC) - 6475 Triple Quadrupole mass spectrometer (UHPLC-MS/MS). The column was Agilent ZORBAX SB-C18 (1.7 μm, 2.1 mm×100 mm); The mobile phases utilized in this study were 0.2% formic acid ( Phase A ) and methanol (Phase B ). The gradient elution procedure was conducted as follows Table S1. The injection volume was set at 5.0 μL, and the flow rate of the mobile phase was maintained at 0.3 mL/min.

The mass spectrometry conditions were as follows: an electrospray ion source operated in positive ion mode (ESI+), the ion source temperature was set at 150 ℃, and multiple reaction monitoring mode (MRM). The accelerating voltage of the collision cell was 4.0 kV, and the desolvation temperature (350 ℃), desolvation gas flow (10 mL/min), and Collision gas flow: 0.15 mL/min.

**Table S1.** The gradient elution procedure

| Elution time/min | Flow rate/mL/min | Phase A volume ratio/% | Phase B volume ratio/% |
| --- | --- | --- | --- |
| 0 (initial) | 0.3 | 90 | 10 |
| 1 | 0.3 | 90 | 10 |
| 2 | 0.3 | 60 | 40 |
| 3 | 0.3 | 20 | 80 |
| 6 | 0.3 | 0 | 100 |
| 8 | 0.3 | 0 | 100 |
| 9 | 0.3 | 90 | 10 |
| 10 | 0.3 | 90 | 10 |

**Table S2.** Information of aquaculture for five sampling ponds

| **Name** | **Main species** | **Aquaculture modes** |
| --- | --- | --- |
| XX | *Tachysurus fulvidraco* (yellow catfish) | In-Pond Raceway System (IPRS) |
| XY | *Tachysurus fulvidraco* (yellow catfish) | Traditional pond |
| XD | *Eriocheir sinensis* (Chinese mitten crab) + M. rosenbergii (freshwater shrimps) | Traditional pond |
| CLD | *Eriocheir sinensis* (Chinese mitten crab) + M. rosenbergii (freshwater shrimps) | Traditional pond |
| THY | *Eriocheir sinensis* (Chinese mitten crab) + M.rosenbergii (freshwater shrimps) | Mulberry-dyke & Fish-pond System |

**Table S3.** Primers and annealing temperature

| **Gene** | **Primer** | **Annealing temperature (^o^C)** | **Amplification Length (bp)** | **Sequence (5’ to 3’)** | **Reference** |  |
| --- | --- | --- | --- | --- | --- | --- |
| *sul*1 | F | 67 | 162 | CGCACCGGAAACATCGCTGCAC | (Jia et al., 2018) |  |
|  | R |  |  | TGAAGTTCCGCCGCAAGGCTCG |  |  |
| *sul*2 | F | 67 | 190 | TCCGATGGAGGCCGGTATCTGG | (Jia et al., 2018) |  |
|  | R |  |  | CGGGAATGCCATCTGCCTTGAG |  |  |
| *tet*A | F | 55 | 210 | GCTACATCCTGCTTGCCTTC | (Zhang et al., 2019) |  |
|  | R |  |  | CATAGATCGCCGTGAAGAGG |  |  |
| *tet*B | F | 55 | 206 | GGTTGAGACGCAATCGAATT | (Jia et al., 2018) |  |
|  | R |  |  | AGGCTTGGAATACTGAGTGTAA |  |  |
| *tet*C | F | 60 | 418 | CTTGAGAGCCTTCAACCCAG | (Jia et al., 2018) |  |
|  | R |  |  | ATGGTCGTCATCTACCTGCC |  |  |
| *tet*G | F | 60 | 468 | GCTCGGTGGTATCTCTGCTC | (Ng et al, 2001) |  |
|  | R |  |  | AGCAACAGAATCGGGAACAC |  |  |
| *tet*M | F | 60 | 60 | CAGAATTAGGAAGCGTGGACAA | (Belén Flórez et al, 2014) |  |
|  | R |  |  | CCTCTCTGACGTTCTAAAAGCGTAT |  |  |
| *tet*W | F | 60 | 60 | ACGGCAGCGCAAAGAGAA | (Belén Flórez et al, 2014) |  |
|  | R |  |  | CGGGTCAGTATCCGCAAGTT |  |  |
| *gyr*A | F | 60 | 330 | AGCGACCTTGCGAGAGAAAT | (Jia et al., 2018) |  |
|  | R |  |  | GGAACCGAAGTTACCCTGACC |  |  |
| *qnr*A | F | 60 | 138 | AGGATTGCAGTTTCATTGAAAGC | (Colomer-Lluch et al, 2014) |  |
|  | R |  |  | TGAACTCTATGCCAAAGCAGTTG |  |  |
| *qnr*B | F | 60 | 134 | CAGATTTYCGCGGCGCAAG | (Jia et al., 2018) |  |
|  | R |  |  | TTCCCACAGCTCRCAYTTTTC |  |  |
| *qnr*S | F | 60 | 118 | CGACGTGCTAACTTGCGTGA | (Colomer-Lluch et al, 2014) |  |
|  | R |  |  | GGCATTGTTGGAAACTTGCA |  |  |
| *erm*B | | F | 60 | 419 | TAACGACGAAACTGGCTAAAATAAG | (Jia et al., 2018) |
|  |  | R |  |  | AACATCTGTGGTATGGCGGG |  |
| *erm*T | | F  R | 60 | 478 | CCGCCATTGAAATAGATCCT  GCTTGATAAAATTGGTTTTTGGA | (Woodbury et al, 2008) |
| *erm*F | | F | 60 | 186 | TCCTTATGGCATTACTTCCGAT | (Jia et al., 2018) |
|  |  | R |  |  | GGACCTACCTCATAGACAAGTTTCA |  |
| *cat*1 | | F | 60 | 132 | CTGGAGTGAATACCACGACGAT | (Jia et al., 2018) |
|  |  | R |  |  | GGATTGGCTGAGACGAAAAAC |  |
| *cmlA* | | F  R | 60 | 234 | TTGGTACGACAGCGAGCACA  AAACAAGGCACGCCGAGG | (Jia et al., 2018) |
| *floR* | | F  R | 60 | 195 | TTTGTCGCTTTCCGTCTACTTC  CTGCCATCCCAAGAACTCG | (Jia et al., 2018) |
| *Inti1* | | F  R | 55 | 280 | CCTCCCGCACGATGATC  TCCACGCATCGTCAGGC | (Zhang et al., 2009) |

**References**

Jia, J., Guan, Y., Cheng, M., Chen, H., He, J., Wang, S., & Wang, Z. (2018). Occurrence and distribution of antibiotics and antibiotic resistance genes in Ba River, China. *The Science of the total environment*, 642, 1136–1144. <https://doi.org/10.1016/j.scitotenv.2018.06.149>

Zhang, C. M., Xu, L. M., Mou, X., Xu, H., Liu, J., Miao, Y. H., Wang, X. C., & Li, X. (2019). Characterization and evolution of antibiotic resistance of Salmonella in municipal wastewater treatment plants. *Journal of environmental management*, 251, 109547. <https://doi.org/10.1016/j.jenvman.2019.109547>

Ng, L. K., Martin, I., Alfa, M., & Mulvey, M. (2001). Multiplex PCR for the detection of tetracycline resistant genes. *Molecular and cellular probes*, 15(4), 209–215. https://doi.org/10.1006/mcpr.2001.0363

Belén Flórez, A., Alegría, Á., Rossi, F., Delgado, S., Felis, G. E., Torriani, S., & Mayo, B. (2014). Molecular identification and quantification of tetracycline and erythromycin resistance genes in Spanish and Italian retail cheeses. *BioMed research international*, 746859. https://doi.org/10.1155/2014/746859

Colomer-Lluch, M., Jofre, J., & Muniesa, M. (2014). Quinolone resistance genes (qnrA and qnrS) in bacteriophage particles from wastewater samples and the effect of inducing agents on packaged antibiotic resistance genes. *The Journal of antimicrobial chemotherapy*, 69(5), 1265–1274. <https://doi.org/10.1093/jac/dkt528>

Woodbury, R. L., Klammer, K. A., Xiong, Y., Bailiff, T., Glennen, A., Bartkus, J. M., Lynfield, R., Van Beneden, C., Beall, B. W., & Active Bacterial Core Surveillance Team (2008). Plasmid-Borne erm(T) from invasive, macrolide-resistant Streptococcus pyogenes strains. *Antimicrobial agents and chemotherapy*, 52(3), 1140–1143. <https://doi.org/10.1128/AAC.01352-07>

Zhang, X., Wu, B., Zhang, Y., Zhang, T., Yang, L., Fang, H. H., Ford, T., & Cheng, S. (2009). Class 1 integronase gene and tetracycline resistance genes tetA and tetC in different water environments of Jiangsu Province, China. *Ecotoxicology* (London, England), 18(6), 652–660. <https://doi.org/10.1007/s10646-009-0332-3>

**Table S4.** Antibiotics detected in this study

| **Antibiotics** | **Abbreviation** | **CAS number** | **Molecular Formula** |
| --- | --- | --- | --- |
| **Sulfonamides (SAs)** |  |  |  |
| Sulfisomidine | SDM | 515-64-0 | C_12_H_14_N_4_O_2_S |
| Sulfamethazine | SMZ | 57-68-1 | C_12_H_14_N_4_O_2_S |
| Sulfadiazine | SDZ | 68-35-9 | C_10_H_10_N_4_O_2_S |
| Sulfathiazole | STZ | 72-14-0 | C_9_H_9_N_3_O_2_S_2_ |
| Sulfapyridine | SPD | 144-83-2 | C_11_H_11_N_3_O_2_S |
| Sulfamerazine | SMR | 127-79-7 | C_11_H_12_N_4_O_2_S |
| Sulfafurazole | SIZ | 127-69-5 | C_11_H_13_N_3_O_3_S |
| Sulfamethoxazole | SMX | 723-46-6 | C_10_H_11_N_3_O_3_S |
| Sulfamono methoxine | SMM | 1220-83-3 | C_11_H_12_N_4_O_3_S |
| Sulfamethizole | SMT | 144-82-1 | C_9_H_10_N_4_O_2_S_2_ |
| Sulfametoxydiazine | SMD | 651-06-9 | C_11_H_12_N_4_O_3_S |
| Sulfamethoxypyridazine | SMXP | 80-35-3 | C_11_H_12_N_4_O_3_S |
| Sulfachloropyridazine | SCP | 80-32-0 | C_10_H_9_ClN_4_O_2_S |
| Phthalylsulfathiazole | PST | 85-73-4 | C_17_H_13_N_3_O_5_S_2_ |
| Sulfadoxine | SDX | 2447-57-6 | C_12_H_14_N_4_O_4_S |
| **Fluoroquinolones (FQs)** |  |  |  |
| Oxilinic acid | OA | 14698-29-4 | C_13_H_11_NO_5_ |
| Ofloxacin | OFL | 82419-36-1 | C_18_H_20_FN_3_O_4_ |
| Norfloxacin | NOR | 70458-96-7 | C_16_H_18_FN_3_O_3_ |
| Ciprofloxacin | CIP | 85721-33-1 | C_17_H_18_FN_3_O_3_ |
| Enrofloxacin | ENR | 93106-60-6 | C_19_H_22_FN_3_O_3_ |
| Flumequine | FL | 42835-25-6 | C14H12FNO3 |
| **Tetracyclines (TCs)** |  |  |  |
| Oxytetracycline | OTC | 79-57-2 | C_22_H_24_N_2_O_9_ |
| Tetracycline | TC | 60-54-8 | C_22_H_24_N_2_O_8_ |
| Chlorotetracycline | CTC | 564-25-0 | C_22_H_24_N_2_O_8_ |
| **Macrolides (MLs)** |  |  |  |
| Tylosin | TYL | 1401-69-0 | C_46_H_77_NO_17_ |
| Clarithromycin | CTM | 81103-11-9 | C_38_H_69_NO_13_ |
| Azithromycin | AZI | 92594-45-1 | C_39_H_74_N_2_O_12_ |
| Roxithromycin | ROX | 80214-83-1 | C_41_H_76_N_2_O_15_ |
| **Chloramphenicol (CAPs)** |  |  |  |
| Florfenicol | FFC | 73231-34-2 | C_12_H_14_Cl_2_FNO_4_S |
| Chloramphenicol | CAP | 56-75-7 | C_11_H_12_Cl_2_N_2_O_5_ |
| Thiamphenicol | TP | 15318-45-3 | C_12_H_15_Cl_2_NO_5_S |
| **Anthelmintic** |  |  |  |
| Thiabendazole | TBZ | 148-79-8 | C_10_H_7_N_3_S |


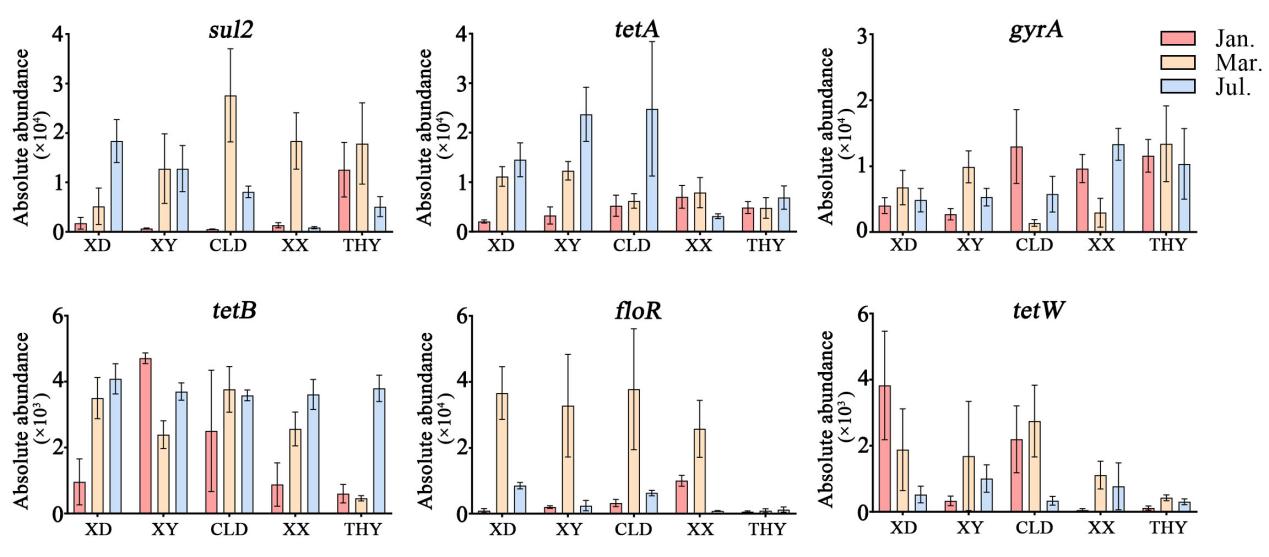


**Figure S1.** Spatiotemporal distribution and variations in absolute abundances of quinolone resistance genes *sul2*, oxytetracycline resistance genes *tetA* and *tetB*, florfenicol resistance genes *gyrA*, and florfenicol-related resistance gene *floR*.


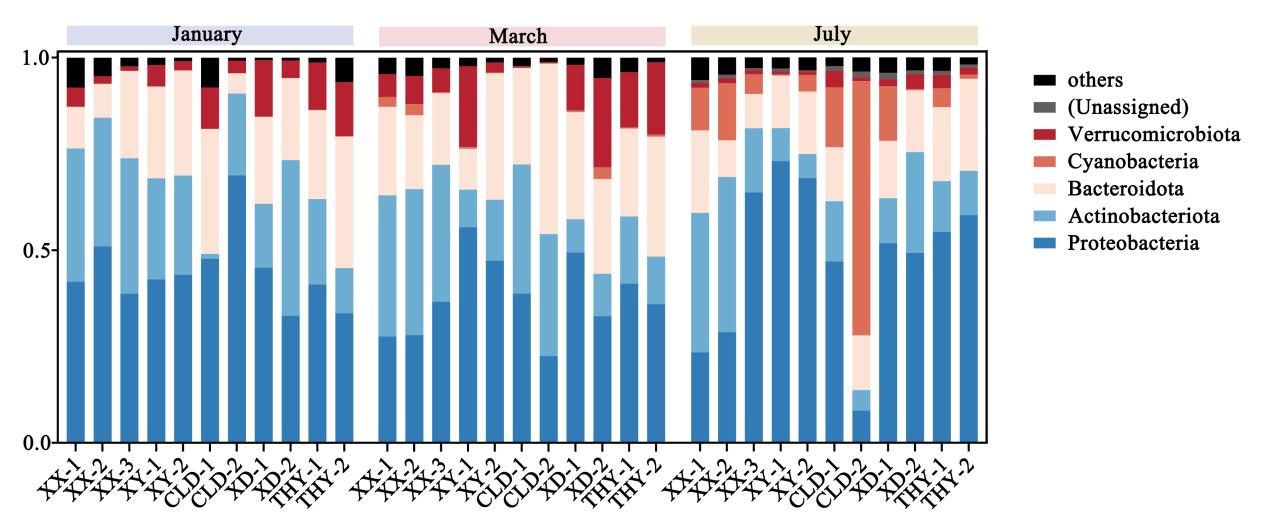


**Figure S2.** The bacterial phyla with the highest relative abundances in each kind of aquaculture ponds in January, March, and October.


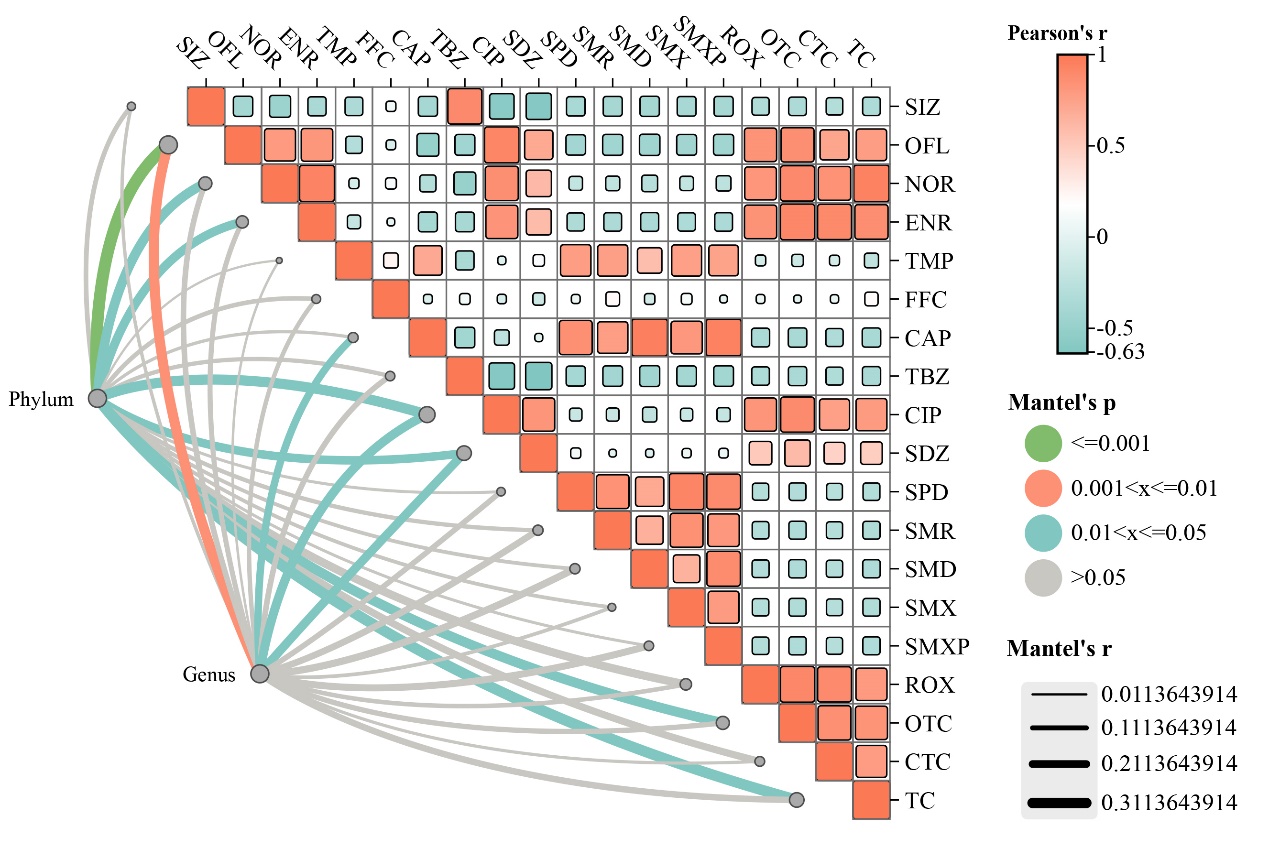


**Figure S3.** The correlation between bacterial community (genus and phylum ) and antibiotics. The heatmap represents the correlation coefficients between different antibiotics. Each box in the heatmap is colored based on the correlation strength, ranging from -0.63 to 1, denoted by a gradient from green to red, red shades are positive, and green is negative. The p-value indicates the significance level, characterized by different colors.

**
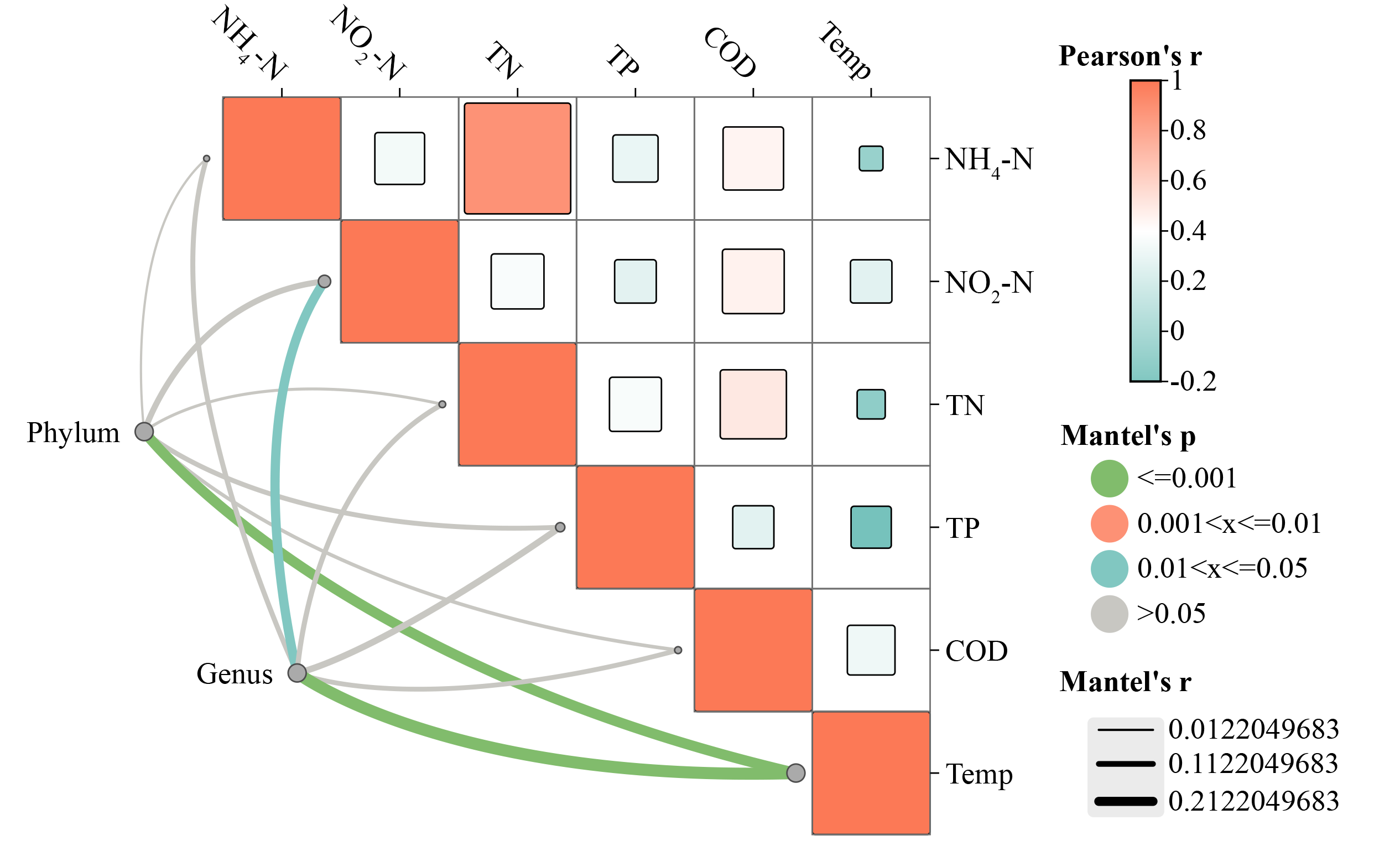
**

**Figure S4.** The correlation between bacterial community (genus and phylum ) and water quality. The parameter settings for the significance analysis are identical to those in Figure S3. NH_4_-N: ammonia; NO_2_-N: nitrite; TN: total nitrogen; TP: total phosphorus; COD: chemical oxygen demand. Temp: water temperature.
